# Establishment of Skeletal Myogenic Progenitors from Non-Human Primate Induced Pluripotent Stem Cells

**DOI:** 10.3390/cells12081147

**Published:** 2023-04-13

**Authors:** June Baik, Carolina Ortiz-Cordero, Alessandro Magli, Karim Azzag, Sarah B. Crist, Aline Yamashita, James Kiley, Sridhar Selvaraj, Ricardo Mondragon-Gonzalez, Elizabeth Perrin, John P. Maufort, Jody L. Janecek, Rachael M. Lee, Laura Hocum Stone, Parthasarathy Rangarajan, Sabarinathan Ramachandran, Melanie L. Graham, Rita C. R. Perlingeiro

**Affiliations:** 1 Department of Medicine, University of Minnesota, Minneapolis, MN 55455, USA; 2 Stem Cell Resources and the Wisconsin National Primate Research Center, University of Wisconsin, Madison, WI 53715, USA; 3 Department of Surgery, University of Minnesota, Minneapolis, MN 55455, USA

**Keywords:** non-human primate, induced pluripotent stem cells, myogenesis, RNA sequencing, muscle regeneration, muscular dystrophy, stem cell therapy

## Abstract

Pluripotent stem (PS) cells enable the scalable production of tissue-specific derivatives with therapeutic potential for various clinical applications, including muscular dystrophies. Given the similarity to human counterparts, the non-human primate (NHP) is an ideal preclinical model to evaluate several questions, including delivery, biodistribution, and immune response. While the generation of human-induced PS (iPS)-cell-derived myogenic progenitors is well established, there have been no data for NHP counterparts, probably due to the lack of an efficient system to differentiate NHP iPS cells towards the skeletal muscle lineage. Here, we report the generation of three independent Macaca fascicularis iPS cell lines and their myogenic differentiation using PAX7 conditional expression. The whole-transcriptome analysis confirmed the successful sequential induction of mesoderm, paraxial mesoderm, and myogenic lineages. NHP myogenic progenitors efficiently gave rise to myotubes under appropriate in vitro differentiation conditions and engrafted in vivo into the TA muscles of NSG and FKRP-NSG mice. Lastly, we explored the preclinical potential of these NHP myogenic progenitors in a single wild-type NHP recipient, demonstrating engraftment and characterizing the interaction with the host immune response. These studies establish an NHP model system through which iPS-cell-derived myogenic progenitors can be studied.

## 1. Introduction

Pluripotent stem (PS) cells represent a valuable option for regenerative medicine, as they provide a virtually unlimited source of lineage-specific progenitors for cell replacement [1]. We have previously demonstrated that the conditional expression of PAX7 enables the generation of large numbers of myogenic progenitors from mouse and human PS cell cultures. Importantly, upon transplantation into different muscular dystrophy mouse models, PS-cell-derived PAX7-induced myogenic progenitors promote muscle regeneration, improve muscle force, and contribute to the resident satellite cell pool [2,3,4,5,6,7]. These encouraging results suggest that PS-cell-derived myogenic progenitors represent a potential therapeutic option for the treatment of skeletal muscle disorders.

Non-human primate (NHP) models play a pivotal role in preclinical studies due to their close similarity to human anatomy and physiology [8]. NHP research not only advances the understanding of numerous pathologies, including cancer, AIDS, Alzheimer’s, Parkinson’s, and diabetes, but also enables researchers to address the safety, efficacy, and mechanism of action of numerous potential therapies [9]. In the specific case of induced PS (iPS)-cell-based therapies, understanding the interaction of engrafted cells with the host immune system is critical for their successful implementation. For instance, recent studies documented the derivation of CD34+ hematopoietic progenitors [10] and retinas [11] from NHP iPS cells for studying the immune response following their transplantation into NHP recipients. Moreover, in the setting of human cell transplants to NHPs, the innate immune system presents species-specific xenogeneic immunological barriers, and thus it is essential to establish a reliable and robust in vitro differentiation protocol for producing tissue-specific NHP iPS cell derivatives to model the clinically relevant allograft situation [12,13,14,15].

Here, we report the generation of skeletal myogenic progenitors from three NHP iPS cell lines. We demonstrate that the specification of the myogenic lineage from NHP iPS cells is associated with global transcriptional changes recapitulating different stages of embryonic development. These progenitors give rise to myotubes in vitro and, more importantly, contribute to muscle regeneration in vivo. Collectively, these findings demonstrate that NHP iPS-cell-derived myogenic progenitors may serve as a highly representative translational model for examining the preclinical aspects of muscle regenerative therapies.

## 2. Material and Methods

### 2.1. iPS Cell Reprogramming, iPS Cell Maintenance, and Myogenic Differentiation

To generate CyMN.1 and CyMN.2 iPS cell lines, CD56+ myoblasts (Supplementary Figure S1) isolated from sterile muscle biopsy were immediately collected after euthanasia from a single donor male Mauritian origin cynomolgus macaque (*Macaca fascicularis*) aged 23.1 years old, weighed 11.54 kg, and with defined MHC haplotype (M3recM2M1). Cells were reprogrammed using a CytoTune™-iPS 2.0 Sendai Reprogramming Kit (Thermo Fisher Scientific, Waltham, MA, USA) under feeder-free conditions, following the manufacturer’s instructions. After the fifth passage, iPS cells were cultured on vitronectin (Gibco, Waltham, MA, USA)-coated dishes using Essential 8 media (Thermo Fischer Scientific) supplemented with 1.94 mg/L of L-Gluthatione (Millipore Sigma, Burlington, MA, USA) and 2.5 µM Endo-IWR1 (Tocris #4423, Minneapolis, MN, USA). The Cy0657#5 iPS cell line was generated from *Macaca fascicularis* p8 fibroblasts at the University of Wisconsin. Reprogramming was completed using a Neon Transfection System 10 μL Kit (Thermo Fisher Catalog #MPK1025). Each reaction consisted of 2 × 10^5^ cells, 0.5 µg each of plasmids ET2K (Addgene #20927, Watertown, MA, USA), EN2L (Addgene #20922), EM2K (Addgene #20923), and miRNA302/367 (Addgene #98748), and 0.5 µg EBNA RNA in 10 µL R Buffer (Thermo Fischer Scientific). Cells were electroporated using the Neon Transfection System Program #4 (1600 V, 20 ms, 1 pulse), and each sample was plated into one well of a six-well matrigel-coated plate in DMEM supplemented with 20% FBS and 1% NEAA (Gibco) for 3–5 days. Cells were then switched to Essential 6 (Life Technologies, Carlsbad, CA, USA) media supplemented with 0.1 µg/mL of human basic fibroblast growth factor (bFGF; PeproTech #100-18) and 100 µM of sodium butyrate for 10 days. After day 10, the cells were maintained in E12 media, consisting of Essential 8 media supplemented with a 1% chemically defined lipid concentrate (Life Technologies, 11905-031), 1% GlutaMAX (Life Technologies, 35050-061), 100 µg/L of nodal (R&D Systems, Minneapolis, MN, USA), and 1.94 mg/L of L-Gluthatione. Colonies were then picked, plated, and maintained on MEFs in E12 and later transitioned to Vitronectin (50 μg/plate).

**Figure 1:**
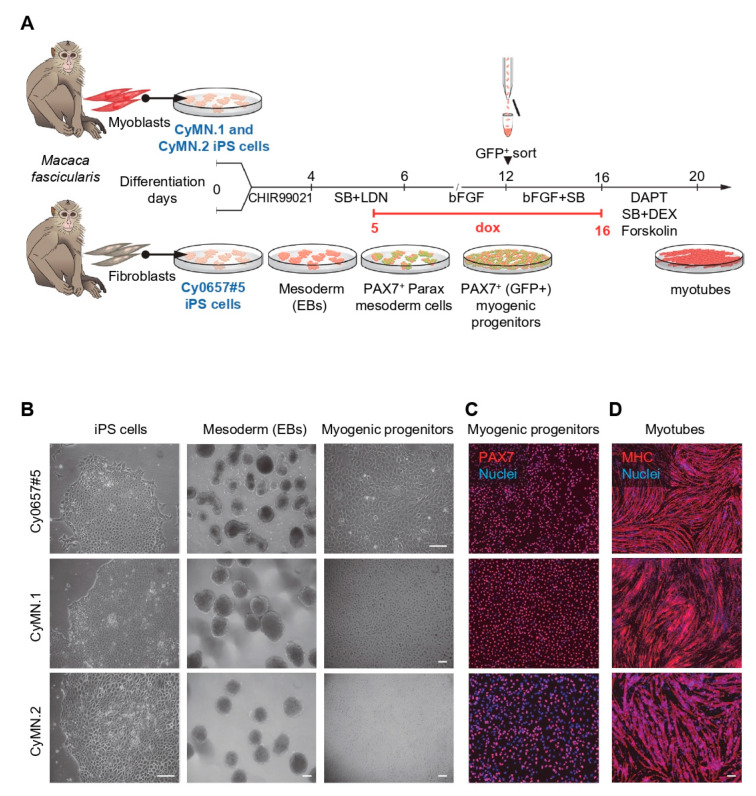
Generation of NHP iPS cells and their in vitro myogenic differentiation: (**A**) Outline of experimental design. PAX7 expression was induced on day 5 of differentiation by adding dox to the medium; (**B**) representative images show the morphology of NHP iPAX7 iPS cell colonies (**left**), day-4 embryoid bodies (EBs; **center**), and expanded PAX7+ myogenic progenitors (**right**); (**C**) immunostaining for PAX7 expression in GFP+-sorted myogenic progenitors from day-12 cultures. PAX7 is shown in red and DAPI stains nuclei in blue; (**D**) representative images show terminal differentiation of PAX7 myogenic progenitors into myotubes. Staining for MHC is shown in red and nuclei in blue. Scale bars, 100 μm.

NHP iPAX7 iPS cells were generated via lentiviral transduction with pCCL-PAX7-ires-GFP and pCCL-rtTA constructs [16]. For the generation of iPAX7 myogenic progenitors, we used the previously reported protocol [17], which is detailed in Figure 1A. On day 2 of the protocol, the medium was replaced with a fresh medium containing 10 µM SB-431542 (Cayman Chemical #13031) and 200 nM LDN-193189 (Cayman Chemical #19396, Ann Arbor, MI, USA). On day 5, the cultures were supplemented with 1 μg/mL doxycycline (dox, Sigma-Aldrich D989, Burlington, MA, USA). On day 12 of the protocol, the cells were sorted using GFP. For expansion, myogenic progenitors were seeded at 0.3–0.4 × 10^6^ per cm^2^ onto gelatin-coated wells in myogenic media consisting of Iscove’s modified Dulbecco’s medium (IMDM) containing 15% fetal bovine serum (FBS), 10% horse serum, 1% KnockOut Serum Replacement™ (KOSR), 1% GlutaMax, 1% penicillin–streptomycin, 50 µg/mL ascorbic acid, and 4.5 mM monothioglycerol, 1 mg/mL dox, 5 ng/mL human bFGF, and 10 µM SB-431542 (Cayman Chemical #13031). Myogenic progenitors were terminally differentiated into myotubes by growing them to confluency and then switching to a terminal differentiation medium, which consisted of DMEM low glucose supplemented with 20% KOSR, 1% insulin–transferrin–selenium, 1% penicillin–streptomycin, 10 µM SB-431542, 10 µM LY-374973 (Cayman Chemical #19396), 10 µM forskolin (Cayman Chemical #11018), and 10 µM dexamethasone (Cayman Chemical #11015) [17]. RFP-labeled NHP iPAX7 CyMN.2 iPS cells were generated by transducing these cells with the lentiviral vector pLKO-H2B-RFP (Plasmid #26001, Addgene). Briefly, lentiviral particles were produced in HEK293T cells by co-transfecting the pLKO-H2B-RFP construct with the packaging plasmids Δ8.9 and pVSVG using a Lipofectamine LTX transfection reagent (Thermo Fisher Scientific). The lentiviral-containing supernatant was collected 48 h after transfection, filtered, and used for the transduction of iPS cell lines upon centrifugation for 1.5 h at 1100× *g* and 37 °C.

For the RNA-sequencing studies of human iPS-cell-derived myogenic progenitors and myotubes, we used the PLZ iPS cell line with conditional expression of PAX7, as previously reported [4]. These cells were differentiated into myogenic progenitors and myotubes using the protocol described by Selvaraj et al. [17], which is similar to the protocol described above for NHP iPAX7 iPS cells.

### 2.2. Teratoma Studies

For teratoma experiments, 1.5 × 10^6^ NHP iPS cells resuspended in 50 μL of 1:1 DMEM/F12:Matrigel (Thermo Fisher Scientific and Corning (Corning, NY, USA), respectively) were injected into the quadriceps of immunodeficient NSG mice (Jackson laboratory, Bar Harbor, ME, USA). Teratomas were collected, fixed, sectioned, and processed for hematoxylin–eosin staining.

### 2.3. Cytogenetic Analysis

G-band karyotype analysis was performed at the Cytogenomics Shared Resource at the Masonic Cancer Center at the University of Minnesota. Live iPS cells were treated with colcemid for 3 h to arrest the cells in metaphase, and 20 different metaphases were analyzed using Giemsa banding at a resolution of 400–450 band level.

### 2.4. RNA Isolation, Library Preparation, and Sequencing

Samples from NHP CyMN.2 and human PLZ iPS cells were resuspended in Trizol (Invitrogen, Waltham, MA, USA) prior to RNA isolation using a PureLinkTM RNA Mini Kit (Invitrogen), following the manufacturer’s instructions for Trizol sample (including in-column DNase treatment). RNAs were submitted for QC and library generation at the University of Minnesota Genomic Center (UMGC). Libraries were generated using a TrueSeq stranded kit and dual-index adapter barcodes at the UMGC. The libraries were then pooled and sequenced on a paired-end run on the NovaSeq (Illumina, San Diego, CA, USA).

### 2.5. Transcriptome Analysis

**Figure 2:**
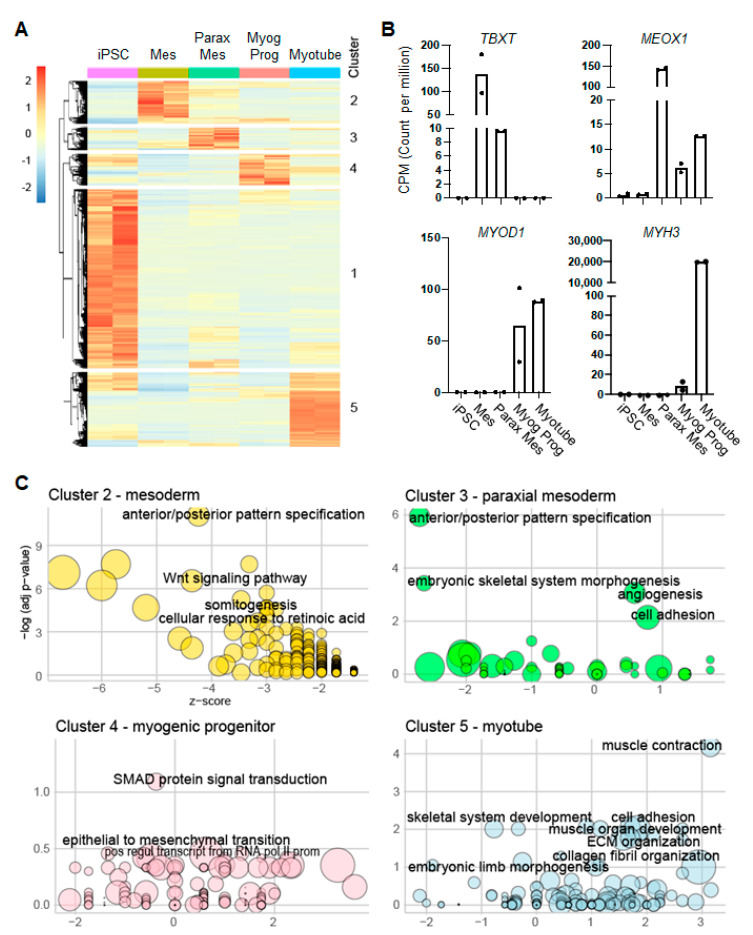
Transcriptional characterization of differentiating NHP iPAX7 iPS cells: (**A**) Heatmap representing unsupervised clustering of differentially expressed transcripts identified during the skeletal myogenic specification of NHP iPAX7 iPS cell lines. Clusters are enumerated from 1 to 5. Pluripotent stem cells: iPSC; uncommitted mesoderm: Mes; paraxial mesoderm: Parax Mes; myogenic progenitors: Myog Prog; myotubes: Myotube; (**B**) expression of selected genes characterizing the clusters identified in panel (**A**). Dots represent replicates; (**C**) functional classification based on gene ontology (GO) analysis using DAVID. Genes from clusters 2 to 5 were analyzed to identify significantly enriched categories, displayed according to −log(adj *p*-value) and z-score. The results are shown as a Bubbleplot generated using the R package GOplot. CPM: counts per million reads.

**Figure 3:**
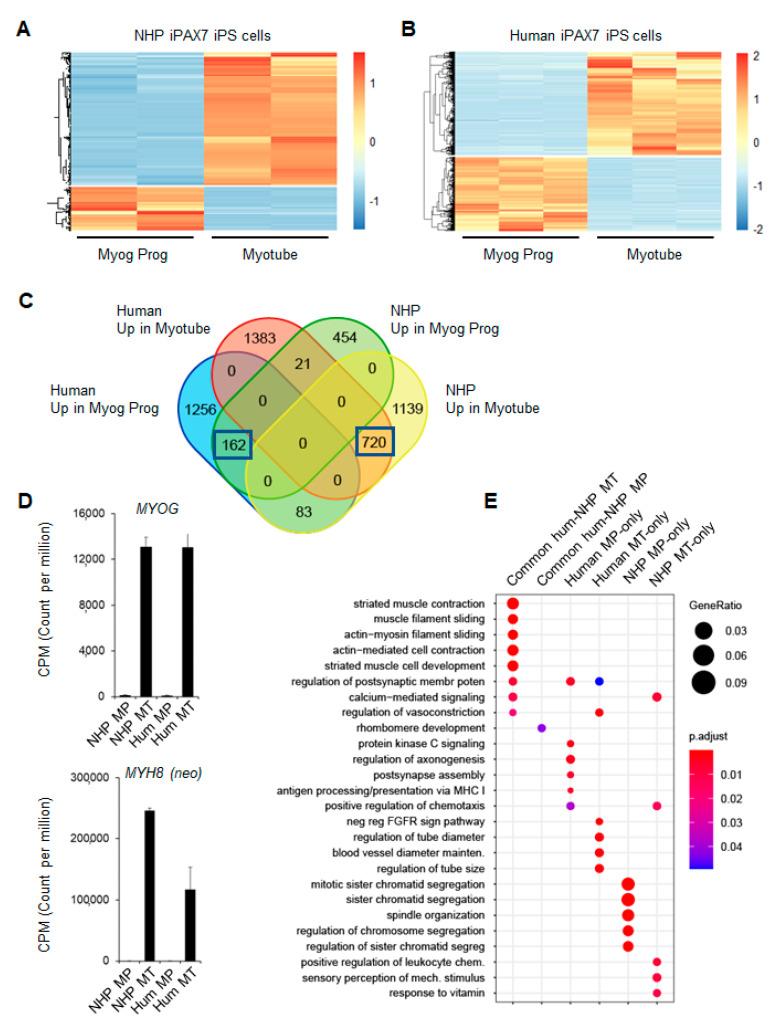
Comparison of NHP and human myogenesis shows high similarity in vitro: (**A**,**B**) Heatmap representing differentially expressed transcripts between myogenic progenitors (Myog Prog) and myotubes (Myotube) derived from NHP and human iPAX7 iPS cell cultures, respectively; (**C**) Venn diagram representing the number of shared genes (blue squares) between human and NHP myogenic cultures; (**D**) expression levels of *MYOG* and *MYH8* in Myog Prog (MP) and Myotube (MT) from NHP and human datasets; (**E**) identification and comparison of biological processes enriched in the different list of genes identified in the Venn diagram from panel (**C**). Gene lists were analyzed using the R package enrichGO. Abbreviation hum-NHP refers to human and NHP. Graph reports the top 5 categories for each cluster. CPM: counts per million reads.

NHP transcriptomic data were mapped to the *Macaca fascicularis* reference genome (macFas6). Human transcriptomic data were mapped to the *Homo sapiens* reference genome (hg38). Data were analyzed using the CHURP pipeline (https://github.com/msi-ris/CHURP) (accessed on 9 April 2020). Differentially expressed transcripts were identified using DESeq2 [18], considering differentially expressed transcripts as all transcripts with q < 0.05 and absolute log2FoldChange > 2. The functional annotation of differentially expressed genes in Figure 2C was performed using the online tool DAVID [19] and plotted using the R package GOplot. The analysis of the functional categories in Figure 3E was performed using the R package clusterProfiler [20] (selecting the “enrichGO” function and “Biological Process” for the ontology).

### 2.6. Transplantation Studies

All animal experiments were carried out according to protocols approved by the University of Minnesota Institutional Animal Care and Use Committee. All animal procedures were executed in an Association for Assessment and Accreditation of Laboratory Animal Care approved facility by qualified staff, following the guidelines and basic principles in the National Institutes of Health (NIH) Guide for the Care and Use of Laboratory Animals [21] the Animal Welfare Act, the U.S. Department of Agriculture, and the U.S. Public Health Service Policy on Humane Care and Use of Laboratory Animals. All animals were purpose-bred and purchased from institutionally approved commercial vendors.

For murine transplantation studies, six-to-eight-week-old NSG (Jackson laboratories) and NSG-FKRP^P448L^ mice were used. One day prior to transplantation, TA muscles were pre-injured with 15–25 µL cardiotoxin 10 µM (Latoxan, Portes-lès-Valence, France). Then, 24 h after cardiotoxin injury, myogenic progenitors were collected from cultures using enzyme-free cell dissociation buffer (Gibco) and resuspended at 1 × 10^6^ in 15 µL of PBS.

For NHP transplantation studies, a single female Mauritian-origin cynomolgus macaque, aged 4.6 years old and weighing 4.36 kg, and with defined MHC (M1 homozygous) was enrolled for transplant. Behavioral management training was employed to eliminate the need for restraint and encourage positive coping behaviors during procedures and testing [22,23]. To realize the need for frequent blood draws while avoiding confounding effects from restraint, sedation, and pain, the recipient was implanted with a vascular access port (VAP), as previously described [24,25]. The recipient was trained to present the port in their homecage as part of a larger cooperative behavioral management program. A standardized diet of 2055C Certified Teklad Global 25% Protein Primate Diet was fed (Envigo, Madison, WI, USA). A standardized enrichment program was used for the duration of the study, including fresh fruits and vegetables, grains, beans, and nuts, as well as a children’s multivitamin.

Animal behavior and clinical status were evaluated at least twice daily. Scheduled physical examinations per protocol and semiannual comprehensive veterinary examinations were performed. Housing was in same-sex pairs, and an environmental enrichment program including social play, toys, music, and regularly scheduled access to a large exercise and swimming area was provided to encourage sensory engagement; enhance foraging behavior and novelty seeking; promote mental stimulation; increase exploration, play, and activity levels; and strengthen social behaviors, increasing the proportion of time spent on species-typical behaviors. Clinical observations included general attitude, activity level, gait, posture, appearance, feces, urine, and any signs of pain or distress. Body weight was measured at least weekly. Sedation for CTX or transplant was achieved using ketamine (5–15 mg/kg IM) and midazolam (0.1–0.3 mg/kg IM), and inhalation isoflurane was used to induce general anesthesia, if necessary. One day prior to cell transplantation, the target muscles were pre-injured with CTX; then, on the day of the transplant, the right quadriceps was intramuscularly injected with 4 × 10^7^ myogenic progenitors using a grid pattern. Blood was collected using the VAP at baseline prior to transplant, and then post-transplant biweekly, weekly, monthly, or bi-monthly based on the phase for clinical pathology that included complete blood counts, blood chemistries, and c-reactive protein levels. The blood concentrations of tacrolimus were measured in whole blood via liquid chromatography/tandem mass spectrometry (M Health Fairview Reference Laboratories, MN). At selected time points, an additional assessment of immune profiles pre- and post-transplant was performed that included antibody response and CD4/CD8 T-cell response, and CD20 B-cell response. At the end of the study, the recipient was euthanized, and a complete necropsy was performed, which included a full gross examination. The harvested tissues were snap-frozen, OCT-embedded, or fixed in 10% neutral-buffered formalin and then embedded in paraffin.

### 2.7. Immunoglobulin Levels

Frozen donor cells were thawed, and after washing with complete RPMI, resuspended at 4 × 10^6^ cells/mL in a FACS buffer (PBS containing 2% FBS). Briefly, 50 µL of prepared donor cell suspension was aliquoted into each well of a U-shape 96-well plate and mixed with 50 µL of recipient serum and incubated at room temperature for 30 min, and then the cells were washed three times in PBS. After the final centrifugation, the cells were resuspended in 100 µL of a FACS buffer containing FITC-anti- IgG, PE-anti-CD20, PE Cy7-anti-CD3, and LIVE/DEAD™ Fixable Aqua dye and incubated at room temperature for 20 min; then, the cells were washed twice and resuspended in the FACS buffer. The stained cells were analyzed using a BD FACS Canto II Flow Cytometer. The detection of anti-IgG or IgM levels on CD3+ or CD20+ gated cells was based on the number of donor-specific antibodies (DSAs) in the recipient’s serum.

### 2.8. Assessment of Donor-Specific T- and B-Cell Proliferation

Single-cell suspensions of lymphocytes from the peripheral blood collected before transplantation (naïve) and after transplant were used for the assessment of donor-specific T- and B-cell proliferation in CFSE-mixed lymphocyte reaction (CFSE-MLR), cell stimulation. Briefly, responder recipients’ lymphocyte samples (600,000 cells) from the recipient monkey were labeled with 1 μM CFSE (Invitrogen, Cat# C34554) and were co-cultured with irradiated (3000 cGy) stimulator myogenic progenitor cells (200,000 cells) in a 1-way CFSE Flow-MLRs for 6 days. For all CFSE-MLR proliferation assays, a 1:3 ratio of responder-to-stimulator cells was maintained. During the flow analysis, the proportion of proliferating CD4+ and CD8+ T cells, as well as CD20+ B cells, was determined based on the percentage of CFSE-negative/low population of gated CD4, CD8, and CD20 lymphocytes.

### 2.9. Immunostaining and Quantification

The cultured cells were washed with PBS and fixed with 4% PFA for 10 min at 4 °C followed by permeabilization with 0.1% Triton X-100 in PBS for 10 min at RT. After washing with PBS, the cells were blocked with 3% BSA in PBS (Thermo Fisher Scientific) for 1 h at RT and then incubated with primary antibody diluted in 3% BSA in PBS overnight at 4 °C. The cells were then washed and incubated with a secondary antibody diluted in 3% BSA supplemented with DAPI for 1 h at RT. After washing with PBS, the cells were maintained in PBS until the final analysis. Images were acquired using an inverted fluorescence microscope (Zeiss, Oberkochen, Germany).

For mouse muscles, upon collection, the muscles were frozen in isopentane cooled in liquid nitrogen, and serial 10–15 μm thick cryosections were collected. Cryosections were then fixed with 4% PFA for 10 min and permeabilized with 0.3% Triton X-100 in PBS for 20 min at RT and then blocked and incubated with primary antibodies overnight. The next day, the slides were washed and incubated with the secondary antibodies, washed again, and were then mounted with coverslips using ProLong Gold Antifade Mountant with DAPI (Thermo Fisher Scientific). The following antibodies were used for immunofluorescence staining: human DYSTROPHIN (MANDYS106, EMD, Burlington, MA, USA); human LAMIN A/C (ab108595, Abcam, Waltham, MA); MHC (MF20, Developmental Studies Hybridoma Bank, Iowa City, IA); OCT3/4 (C-10, Santa Cruz Biotechnology, Dallas, TX, USA); SOX2 (Y-17, Santa Cruz Biotechnology); NANOG (H-2, Santa Cruz Biotechnology); Alexa Fluor 488 anti-rabbit IgG (A-11008, Thermo Fisher Scientific); Alexa Fluor 647 goat anti-mouse IgG (A-21235, Thermo Fisher Scientific); and Alexa Fluor 555 goat anti-mouse IgG (A21424, Thermo Fisher Scientific).

For NHP, upon necropsy, muscles of interest were collected, immediately mounted in OCT Compound (Sakura Finetek Japan. Co., Ltd., Tokyo, Japan), and cooled in liquid nitrogen. Serial 14 μm thick cryosections were collected. The cryosections were fixed with 4% PFA for 30 min and then permeabilized with 0.3%Triton X-100 in PBS for 15 min at RT. The slides were blocked with 3% BSA for 30 min at RT and then stained with rabbit RFP (ab62341, Abcam) and mouse DYSTROPHIN (DYS1-CE, Leica, Nanterre Cedex, France) at 4 °C overnight. Sections were washed with PBS and incubated with Alexa Fluor 647 goat anti-mouse IgG (A-21235, Thermo Fisher Scientific) and Alexa Fluor 555 goat anti-rabbit IgG (A-21428, Thermo Fisher Scientific) for 1 h at RT, washed again, and were then mounted with coverslips using ProLong Gold Antifade Mountant with DAPI (Thermo Fisher Scientific).

### 2.10. qPCR

For sensitivity testing, unlabeled (non-RFP) CyMN.2 NHP myogenic progenitors and H2B.RFP-labeled CyMN.2 myogenic progenitors were grown in culture until sufficient cell numbers were generated. H2B.RFP-labeled CyMN.2 cells were mixed with unlabeled CyMN.2 such that the following dilutions were made: 1:1, 1:10, 1:10^2^, 1:10^3^, 1:10^4^, 1:10^5^, and 1:10^6^. Genomic DNA was then extracted using Qiagen’s DNeasy Blood and Tissue Kit (69504). At this time, the H2B.RFP-labeled CyMN.2 sample was also diluted ten-fold in series to create a standard curve. qPCR reactions were carried out using the DNA samples (100 ng per reaction), a TaqMan Gene Expression Master Mix (4369016, ThermoFisher), and amplicon-specific primer/probe sets (RFP and primate-specific AluYB8 (internal control), Invitrogen). RFP-Fw: GCC CTT CGC CTG GGA CAT, RFP-Rv: GGT GCT TCA CGT ACA CCT TGG A, RFP-Probe: 6-FAM-CTG TCC CCC CAG TTC CAG TAC GG- TAMRA. AluYB8-Fw: GTC AGG AGA TCG AGA CCA TCCT, AluYB8-Rv: AGT GGC GCA ATC TCG GC, AluYB8-Probe: 6-FAM-AGC TAC TCG GGA GGC TGA GGC AGG A-TAMRA. Samples were run in at least triplicate on a QuantStudio 6 Flex Real-Time PCR system (Applied Biosystems, Waltham, MA, USA) using the following cycling conditions: (1) 50 °C for 2 min; (2) 95 °C for 10 min; (3) 95 °C for 15 s, followed by 61 °C for 1 min repeated for 39 more cycles; and (4) 61 °C for 3 min. RFP Ct values were normalized by AluYB8 Ct values. Additional details are provided in Supplementary Table S1.

For NHP tissue, upon necropsy, muscles of interest were immediately flash-frozen (LN_2_). Genomic DNA was prepared as described above. qPCR reactions were carried out using the DNA samples (100 ng per reaction), TaqMan Gene Expression Master Mix (4369016, ThermoFisher), and amplicon-specific primer/probe sets (RFP and primate-specific AluYB8 (internal control), Invitrogen). RFP and AluYB8 primers are described above. The samples were run in at least triplicate using the same cycling conditions described above. The ∆∆Ct method was used, where the Ct value of RFP was normalized to the assay’s internal loading control (e.g., ∆Ct (RFP-AluYB8)), followed by the normalization of the transplanted muscles to that of non-transplanted (e.g., ∆Ct (transplanted—control)). In these studies, the diaphragm was used as the non-transplanted negative control. The biceps received an intramuscular saline injection but no delivery of cellular therapy, so they were also considered a negative control. The quadriceps received an intramuscular injection of the H2B.RFP-labeled CyMN.2 myogenic progenitors. Additional details are provided in Supplementary Table S2.

### 2.11. Statistical Analysis

Differences between samples were assessed using an unpaired one-tailed Student’s *t*-test or two-way ANOVA for multiple comparisons followed by Tukey’s multiple-comparison tests. *p* values < 0.05 were considered significant.

### 2.12. Data Availability

Raw and processed RNA-seq data were deposited in the NCBI Gene Expression Omnibus (GEO) database under accession code GSE228567.

## 3. Results

### 3.1. PAX7 Induces Myogenic Specification of NHP iPS Cells

To investigate our ability to induce skeletal myogenesis from NHP iPS cells, we assessed three independent NHP iPS cell lines generated from *Macaca fascicularis*: CyMN.1, CyMN.2, and Cy0657#5 (Figure 1A). CyMN.1 and CyMN.2 were reprogrammed from myoblasts (Supplementary Figure S1), whereas Cy0657#5 was reprogrammed from fibroblasts. All three NHP iPS cell lines displayed typical pluripotent stem cell morphology (Figure 1B, left panels), and characterization confirmed the expression of pluripotency markers (Supplementary Figure S2A), normal karyotypes (Supplementary Figure S2B), and their ability to develop teratomas containing all three germ layers upon their injection into immunodeficient mice (Supplementary Figure S2C).

To induce the skeletal muscle fate, we transduced NHP iPS cell lines with lentiviral vectors harboring a doxycycline (dox)-inducible PAX7-ires-GFP expression system (hereafter referred to as NHP iPAX7 iPS cells), and these cells were then subjected to a multistep in vitro differentiation protocol, as outlined in Figure 1A. All NHP iPAX7 iPS cell lines efficiently formed embryoid bodies (EBs) (Figure 1B). Upon PAX7 induction and subsequent FACS-mediated purification of PAX7+ (GFP+) progenitors on day 12, the resulting cultures expressed PAX7 (Figure 1B,C). Upon dox withdrawal, iPAX7 myogenic progenitors from all three NHP iPAX7 iPS cell lines underwent terminal myogenic differentiation, as indicated by the presence of myosin heavy chain (MHC)-expressing myotubes (Figure 1D). These data support the ability of NHP iPAX7 iPS cells to differentiate into skeletal myogenic progenitors and subsequently into myotubes in vitro.

### 3.2. Global Transcriptional Characterization of Differentiating NHP iPAX7 iPS Cells

The skeletal myogenic specification is a multistep process controlled by the concerted action of signaling pathways and transcription factors [26]. While these mechanisms have been investigated in murine and human systems [27,28,29], studies involving NHP myogenic cells have so far mainly focused on the in vivo regenerative potential of adult myoblasts [30,31]. To characterize the developmental processes associated with the myogenic commitment of NHP iPAX7 iPS cells, we next performed a whole-transcriptome analysis at different stages of the differentiation protocol using the CyMN.2 iPS cell line (Figure 1A): pluripotent stem cells (iPSC—day 0), mesodermal cells (Mes/EBs—day 4), paraxial mesoderm cells (Parax Mes—day 6), PAX7+ proliferating myogenic progenitors (Myog Prog—day 13), and differentiated myotubes (Myotube—day 20). RNA sequencing (RNA-seq) identified 2724 differentially expressed genes whose expression levels displayed a fold change > 2 and a false discovery rate (FDR) of < 0.05. Principal component analysis separated these time points based on their expected developmental trajectory (Supplementary Figure S3A) and, as shown in Figure 2A, unsupervised analysis of these differentially expressed genes revealed five main clusters. The genes specific for each cluster included *NANOG* (Cluster 1), *BRACHYURY* (*TBXT*—Cluster 2), *MEOX1* (Cluster 3), *MYOD1* (Cluster 4), and *MYH3* (Cluster 5), which represent well-known markers associated with pluripotency, uncommitted mesoderm, paraxial mesoderm, myogenic progenitors and myotubes, respectively (Figure 2B and Supplementary Figure S3B). Importantly, the gene ontology (GO)-based functional annotation of each cluster revealed enriched biological processes known to be associated with the transition from uncommitted mesoderm to skeletal muscle, as exemplified by the WNT signaling pathway (Cluster 2), anterior–posterior pattern specification (Cluster 3), epithelial-to-mesenchymal transition (Cluster 4), and muscle organ development (Cluster 5) (Figure 2C). Together, these data demonstrate that the specification of the skeletal myogenic lineage using NHP iPAX7 iPS cells is associated with global transcriptional changes, recapitulating key molecular changes described during embryogenesis in model organisms.

### 3.3. Transcriptional Convergence of the Myogenic Programs between NHP and Human iPS-Cell-Derived Cultures

The translational potential of human iPS-cell-derived myogenic progenitors has prompted multiple efforts toward the identification of surface markers that could be used for the prospective isolation of these cells from mixed cultures: CXCR4 and MET [32]; CD54 (also referred to as ICAM1), ITGA9, and SDC2 [16]; ERBB3 and NGFR [33]; CD10 and CD24 [34]; CDH13 and FGFR4 [35]; and NCAM1 (also referred to as CD56) [36]. In support of the similarities between the NHP and human iPS-cell-based systems, the inspection of our transcriptomic dataset evidenced that, among these markers, *CD54*, *SDC2*, *CDH13*, and *MET* showed higher expression at the myogenic progenitor stage (Supplementary Figure S3C). While we previously demonstrated that CD54 enables the purification of human progenitors [16], we could not use this approach due to the lack of an antibody cross-reacting with *M. fascicularis* CD54. Since both CD54 and SDC2 have been previously reported to identify human iPS-cell-derived iPAX7+ myogenic progenitors generated using the PAX7-inducible differentiation protocol [16], we speculated that the comparable expression profiles of these markers might reflect a global similarity between NHP and human molecular programs.

To provide an unbiased comparison between these two cell populations, we next performed a global comparison of NHP and human PS-cell-derived myogenic cells (at the myogenic progenitor and myotube stages) using RNA-seq. As shown in Figure 3A,B, transcriptomic analyses identified several differentially expressed genes between the myogenic progenitor and the myotube stages in each individual cell line (2579 and 3624 differential transcripts in NHP and human cells, respectively). The intersection of these two datasets evidenced a substantial overlap between NHP and human myogenic cells: 162/637 (25%) NHP genes at the myogenic progenitor stage and 720/1942 (37%) NHP genes at the myotube stage (Figure 3C). As expected, the list of shared NHP and human myotube transcripts included the genes involved in biological processes related to skeletal muscle development and function, as exemplified by *MYOG* and *MYH8* (Figure 3D,E). While this analysis identified gene sets with a concordant or discordant expression between NHP and human myogenic cells, these data support that the iPAX7-mediated myogenic commitment of NHP iPS cells recapitulates a subset of the molecular features characterizing human iPS-cell-derived myogenic cells.

### 3.4. In Vivo Regenerative Potential of NHP iPAX7 Myogenic Progenitors

**Figure 4:**
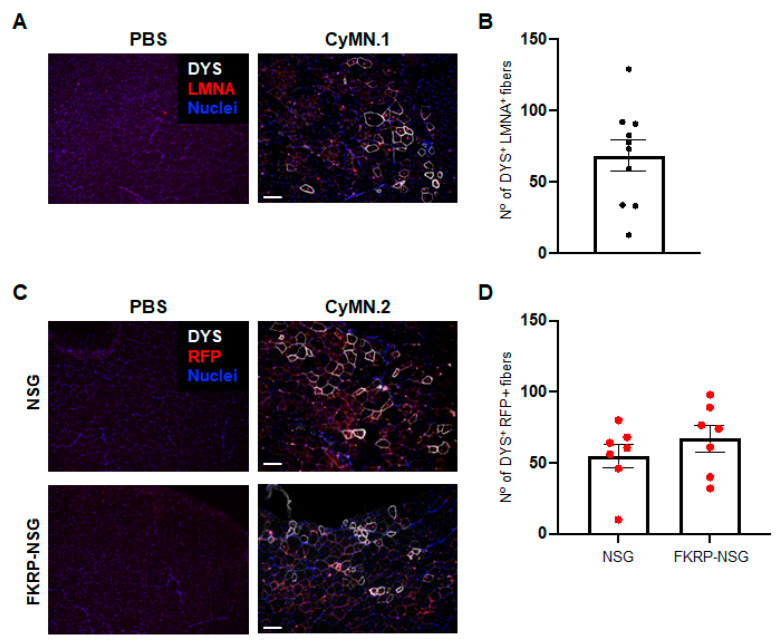
NHP iPAX7 iPS-cell-derived myogenic progenitors contribute to in vivo muscle regeneration in mice: (**A**,**B**) In vivo regenerative capacity of CyMN.1 iPAX7 myogenic progenitors in mice: (**A**) Representative engraftment resulting from intramuscular transplantation of CyMN.1 iPS-cell-derived myogenic progenitors in immunodeficient (NSG) mice. PBS-injected muscle served as a negative control. Injected muscles were analyzed using primate-specific antibodies for DYSTROPHIN (DYS—white) and LAMIN A/C (red). Nuclei (blue). Scale bar is 100 µm; (**B**) quantification of muscle fiber engraftment. Dots represent average engraftment on each TA and bars represent mean ± standard error. CyMN.1 (n = 5, 10 TAs); (**C**,**D**) donor-derived RFP-labeled CyMN.2 PAX7+ myogenic progenitors are identified in the CTX-injured TA muscles of mice. PBS-injected muscles served as a negative control: (**C**) Transplanted muscles were analyzed using primate-specific antibodies for DYSTROPHIN (DYS—white) and RFP (red). Nuclei (blue). Scale bar is 100 µm; (**D**) quantification of myofiber engraftment. Dots represent each TA and bars represent mean ± standard error. NSG (n = 7) and FKRP-NSG (n = 7).

An important feature of human iPS-cell-derived iPAX7 myogenic progenitors is their ability to contribute to skeletal muscle regeneration following intramuscular injection. To determine the in vivo regenerative potential of NHP counterparts, we transplanted CyMN.1 iPS-cell-derived iPAX7 myogenic progenitors into cardiotoxin (CTX)-injured tibialis anterior (TA) muscles of immunodeficient (NSG) mice. At 6–8 weeks post-transplantation, we detected the presence of NHP donor-derived myofibers (Figure 4A,B) using antibodies recognizing LAMIN A/C (LMNA) and DYSTROPHIN (DYS).

A similar outcome was observed when we transplanted CyMN.2 iPS-cell-derived iPAX7 myogenic progenitors into NSG mice (Figure 4C, upper panel). Considering our plan to transplant these cells into an NHP recipient, to distinguish donor-derived engraftment, we labeled CyMN.2 iPAX7 iPS cells with a lentiviral vector encoding the nuclear-localized H2B-RFP fusion protein (hereafter referred to as RFP), which we have previously demonstrated to give robust expression in mouse donor-derived myofibers [3,37]. The transplantation of RFP-labeled CyMN.2 PAX7+ myogenic progenitors into the CTX-injured TA muscles of NSG as well as of FKRP-NSG mice, an immunodeficient mouse model of limb-girdle muscular dystrophy R9 [3], resulted in comparable engraftment, as shown by the presence of donor-derived DYS+ myofibers with RFP+ nuclei (Figure 4C,D).

**Figure 5:**
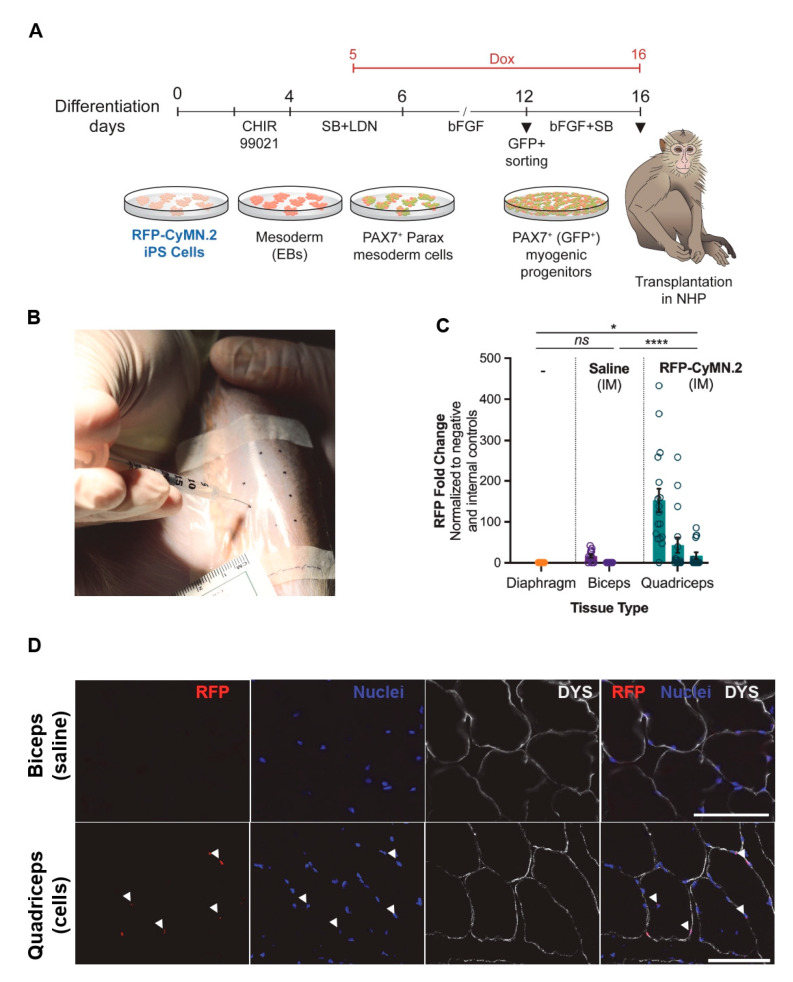
NHP iPAX7 iPS-cell-derived myogenic progenitors show engraftment capacity in NHP: (**A**) Schematic representation of the derivation of myogenic progenitors and transplantation in an NHP; (**B**) cells were suspended and injected IM at CTX-pre-injured sites using a grid pattern; (**C**,**D**) donor-derived RFP-labeled CyMN.2 iPAX7 myogenic progenitors are identified in the quadriceps of NHP recipient using qPCR and immunofluorescent assays: (**C**) Dot plot depicting fold change in RFP signal detected in skeletal muscle sites collected upon necropsy, where diaphragm represents an unmanipulated negative control. Biceps received an intramuscular injection of saline (negative control), whereas the quadriceps received an intramuscular injection of 4 × 10^7^ RFP-labeled CyMN.2 myogenic progenitors. Each bar represents a separate muscle biopsy collected at necropsy. Each dot represents one technical replicate. n = 3 independent replicates, with 3–5 technical replicates each. Two-way ANOVA, with Tukey’s multiple-comparison tests, where * *p* = 0.0307 for diaphragm *v*. quad, **** *p* < 0.0001 for biceps *v*. quad. Diaphragm *v.* biceps was n.s. (*p* = 0.43). Error bars depict the mean with standard error of the mean (s.e.m) for all; (**D**) representative images show muscle section from saline-injected biceps muscle of NHP recipient (upper panel). Donor-derived RFP+ nuclei (RFP; red) counterstained with DAPI (DAPI; blue) and DYSTROPHIN (DYS—white) myofibers are identified as donor-derived RFP-labeled CyMN.2 PAX7+ myogenic progenitors in muscle section from quadriceps muscle of NHP recipient (lower panel). White arrowheads indicate RFP+DAPI+ nuclei. (Scale bar, 100 μm).

Having validated the in vivo regenerative potential of RFP-labeled NHP PAX7+ myogenic progenitors in mouse models, we next tested the ability of these cells in contributing to muscle regeneration in an NHP recipient (Figure 5A). After confirming a favorable MHC crossmatch by screening for the absence of donor-specific antibodies (Supplementary Figure S4A), we immunosuppressed a single recipient with tacrolimus monotherapy at therapeutic levels (measured at 10.4 ± 4.8 μg/mL). The quadriceps muscle was injured via CTX injection, and 24 h later, 4 × 10^7^ cells were injected intramuscularly at multiple sites (Figure 5B). A volume-matched saline control was injected into the CTX-injured biceps muscle of the same recipient. The presence of anti-donor antibodies was evaluated at baseline and post-transplantation; a three-fold sustained increase in the levels of IgG antibodies was observed, with no appreciable increase in the levels of IgM antibodies (Supplementary Figure S4B). To evaluate anti-donor T-cell responses, we analyzed the proliferation of donor-specific CD4+ and CD8+ T cells and CD20+ B cells in a one-way carboxyfluorescein diacetate succinimidyl ester (CFSE) mixed lymphocyte reaction (MLR) cultures at baseline and post-transplantation using PBMCs stimulated with irradiated NHP-origin myogenic progenitor cells. At baseline, the recipient met the threshold of pre-existing donor-specific immunity (≤5% proliferation). The donor antigen-specific proliferation of CD4 and CD20 remained near baseline, while CD8 increased two-fold by day 56 and remained elevated through the study follow-up period until day 64 (Supplementary Figure S4C). The tolerability of RFP-labeled NHP PAX7+ myogenic progenitors was examined in the NHP recipient via clinical observations, complete blood counts, and serum chemistries, with a complete necropsy at the end of the study. Body weights were stable with a gain trend, and there was no change in the serum C-reactive protein, an acute inflammation marker. Complete blood counts remained within normal limits throughout the follow-up. At 8 weeks post-transplantation, we evaluated engraftment using quantitative real-time PCR (qPCR) and immunofluorescence. After confirming the sensitivity of the RFP signal using qPCR (Supplementary Figure S5A–C), we subjected the DNA samples collected from the NHP recipient to qPCR. As shown in Figure 5C, we observed significant RFP expression in the transplanted quadriceps, while this was negligible in the biceps and diaphragm control muscles (PBS-injected and untreated, respectively). In agreement with the qPCR results, we observed the presence of donor-derived RFP+ nuclei in DYS+ myofibers in the transplanted quadriceps (Figure 5D and Supplementary Figure S5D). Together, these data support the feasibility of using NHP iPAX7 iPS-cell-derived myogenic progenitors in studies to model iPS-cell-based myogenic cell therapy approaches in vivo.

## 4. Discussion

Although NHP iPS cells were first documented in 2008, when Liu and colleagues established iPS cells from adult rhesus fibroblasts [38], only more recently has the field witnessed a flourish of studies on the in vitro derivation of lineage-specific cell types from NHP iPS cells. These include the generation of cardiomyocytes [39], neurons [40], cartilage [41], corneal epithelial sheets [42], and retinas [11], as well as hematopoietic progenitors [10], which have been mostly used to study engraftment and immune response. This has yet to be investigated in the context of skeletal muscle regeneration and muscular dystrophies due to the lack of knowledge in NHP myogenesis and protocols to generate iPS cell-skeletal myogenic derivatives.

Here, we report for the first time the establishment of an in vitro differentiation system enabling the derivation of skeletal myogenic progenitors from NHP iPS cells. These progenitors give rise to terminally differentiated myotubes in vitro and contribute to muscle regeneration in vivo. We leverage this system to investigate the transcriptomic changes characterizing the specification of *M. fascicularis* mesodermal progenitors toward the skeletal myogenic lineage, and to identify the similarities between the molecular programs controlling myogenesis in human and non-human primates. Our findings demonstrating that NHP iPS-cell-derived myogenic progenitors are capable of contributing to muscle regeneration in both murine and NHP models provide feasibility for future studies aimed at assessing the immune response as well as the safety of iPS-cell-derived myogenic progenitors.

The use of large animal models for the preclinical assessment of cell-based muscle regenerative therapies has so far focused on the transplantation of adult cells, such as myoblasts in NHP [31,43,44,45,46] and mesoangioblasts in dog [47] recipients. These studies provide important insights into the clinical translatability of these cell types and indicate the necessity of better understanding the interaction between donor cells and the immune system [45,48]. Because the major histocompatibility complex (MHC) of Mauritian cynomolgus macaques (MCMs) is fully characterized and with similar diversity to the clinical situation [49,50], the iPS cell system developed here will be valuable to determine the influence of MHC on the short- and long-term engraftment of iPS-cell-derived myogenic progenitors. For instance, our findings on the transplantation of CyMN.2 myogenic progenitors in the NHP recipient show a two-fold increase in IgG (Supplementary Figure S4B) together with increased CD8+ proliferating cells (Supplementary Figure S4C) in the post-transplant phase, suggesting that the immunosuppressive regimen may benefit from further refinement. Tacrolimus monotherapy is generally not sufficient to persistently suppress immune responses against transplanted tissue, typically resulting in mean graft survival of less than 3 months [51]. The injection of RFP-labeled NHP PAX7+ myogenic progenitors was well tolerated; however, we observed a similar pattern of rejection to what has been previously reported with tacrolimus alone where chronic rejection results from the activation of cellular and humoral immune response (Supplementary Figure S4B,C) and gradual loss of transplanted cells. Nevertheless, we established robust NHP iPS-cell-derived myogenic progenitors and a homologous model system, functionalizing the development of allogeneic and autologous transplantation and enabling the fine-tuning of the corresponding immunosuppression regimens prior to human trials.

The NHP iPS cell myogenic differentiation method described here provides feasibility for the use of NHP myogenic progenitors for in vitro muscle systems and in vivo cell transplantation. Future studies will enable the understanding of the impact of MHC mismatch on muscle engraftment as well as guide safe and efficacious delivery routes, which will further contribute to the development of an optimized iPS-cell-based therapy for muscular dystrophy patients.

## Data Availability

For any additional questions, please contact the corresponding author.

## Supplementary Materials

The following supporting information can be downloaded at: https://www.mdpi.com/article/10.3390/cells12081147/s1, Figure S1: Derivation of myoblasts from NHP muscle biopsies; Figure S2: Characterization of NHP iPS cell lines; Figure S3: Transcriptomic profiling of differentiating NHP iPAX7 iPS cells; Figure S4: Pre- and post-transplant immune monitoring; Figure S5: Sensitivity test of RFP signal for RFP-labeled CyMN.2 PAX7+ myogenic progenitors in NHP recipient.
