# Supplementary material for: Establishment of Skeletal Myogenic Progenitors from Non-Human Primate Induced Pluripotent Stem Cells
Source: Cells. 2023 Apr 13;12(8):1147. doi: 10.3390/cells12081147 (PMC10137227; doi:10.3390/cells12081147)
Supplement: Supplementary file 1 [file cells-12-01147-s001.zip › cells-2274837-supplementary.pdf]

SUPPLEMENTARY FIGURE LEGENDS

Supplementary Figure S1

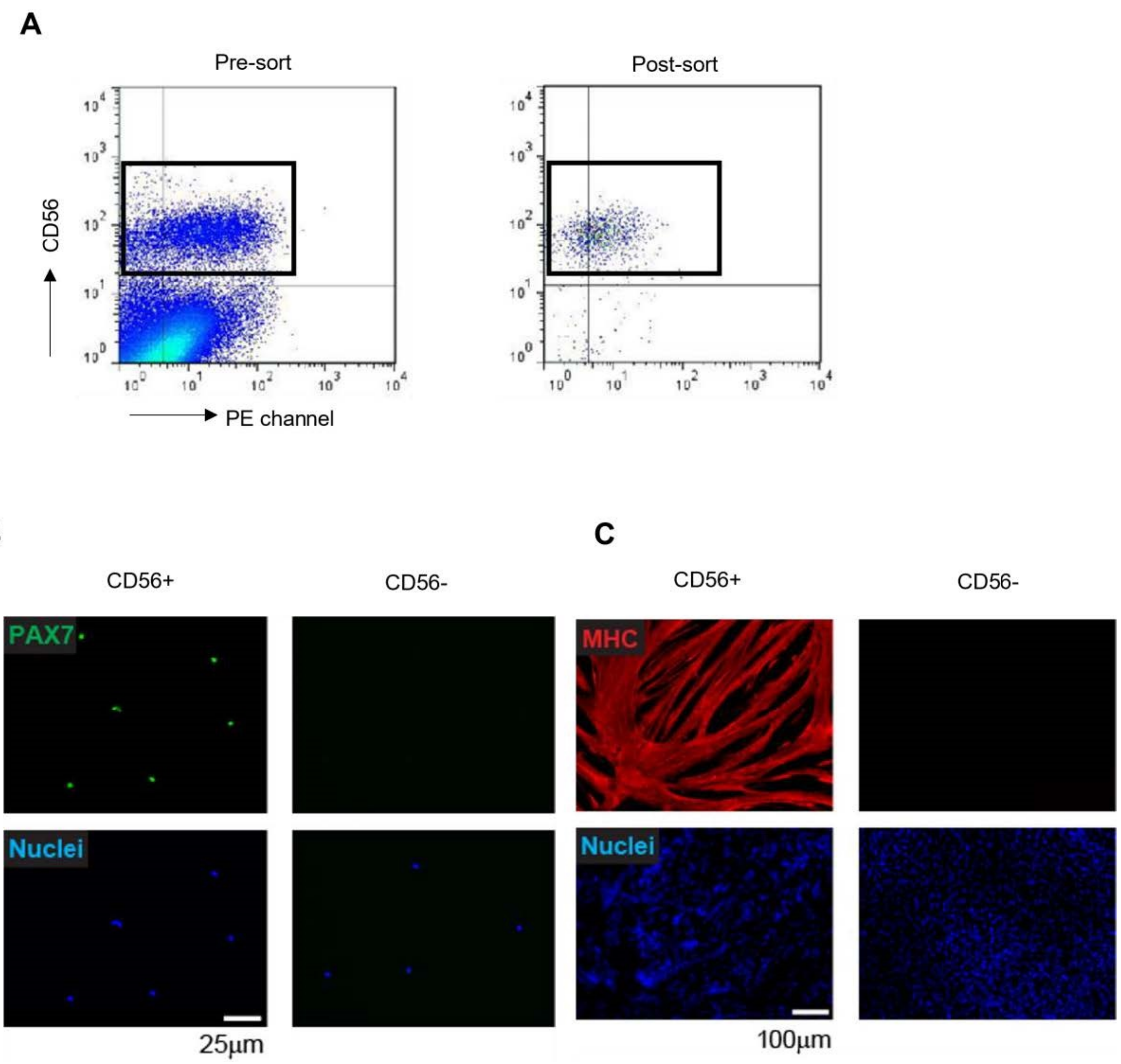

Supplementary Figure S1. Derivation of myoblasts from NHP muscle biopsies. A) Identification and purification of NHP satellite cells using FACS in the mononuclear fraction derived from the NHP quadriceps biopsy. Pre-sort (left panel) and post-sort (right panel) cytometry data report the expression of CD56 (y-axis) and CD82 (x-axis). NHP satellite cells were identified based on the expression of CD56 (black box). CD82 was not used for the purification. Post-sort analysis (right panel) confirms enrichment for the CD56+ fraction. B) Immunostaining shows PAX7 expression only in the

CD56+ cell fraction. PAX7 is shown in green and DAPI stains nuclei in blue. Scale bar: 50μm. C) Only CD56+ *in vitro* expanded cells show the ability to terminally differentiate into myotubes in vitro. Images show immunostaining for MHC in red and nuclei in blue. Scale bar: 100μm.

Supplementary Figure S2

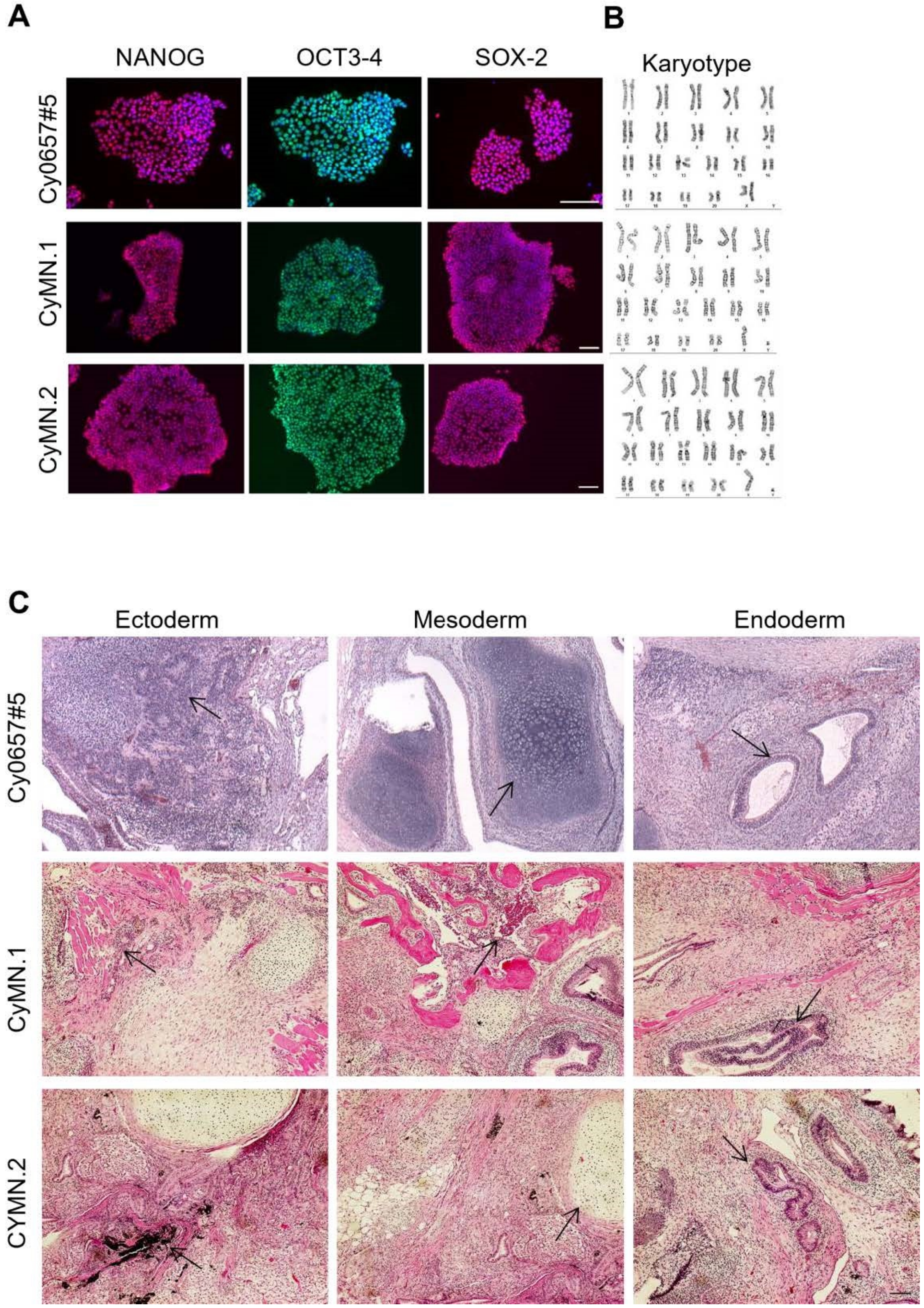

Supplementary Figure S2. Characterization of NHP iPS cell lines. A) Expression of NANOG, OCT3-4, and SOX-2 in NHP iPS cell lines. B) Cytogenetic analyses. C) Representative images show H&E

staining of developed teratomas upon the injecting of Cy0657#5, CyMN.1, and CyMN.2 NHP iPS cell lines in the quadriceps of NSG mice. Teratomas were collected ~8 weeks post-injection. Images show the ability of these iPSC clones to contribute to all three germ layers. In the ectoderm images, arrows in the upper and middle panels point to neuroectoderm, while arrow in the lower panel points to pigmented epithelium. In the mesoderm images, arrows point to cartilage. In the endoderm images, arrows points to ciliated epithelium.

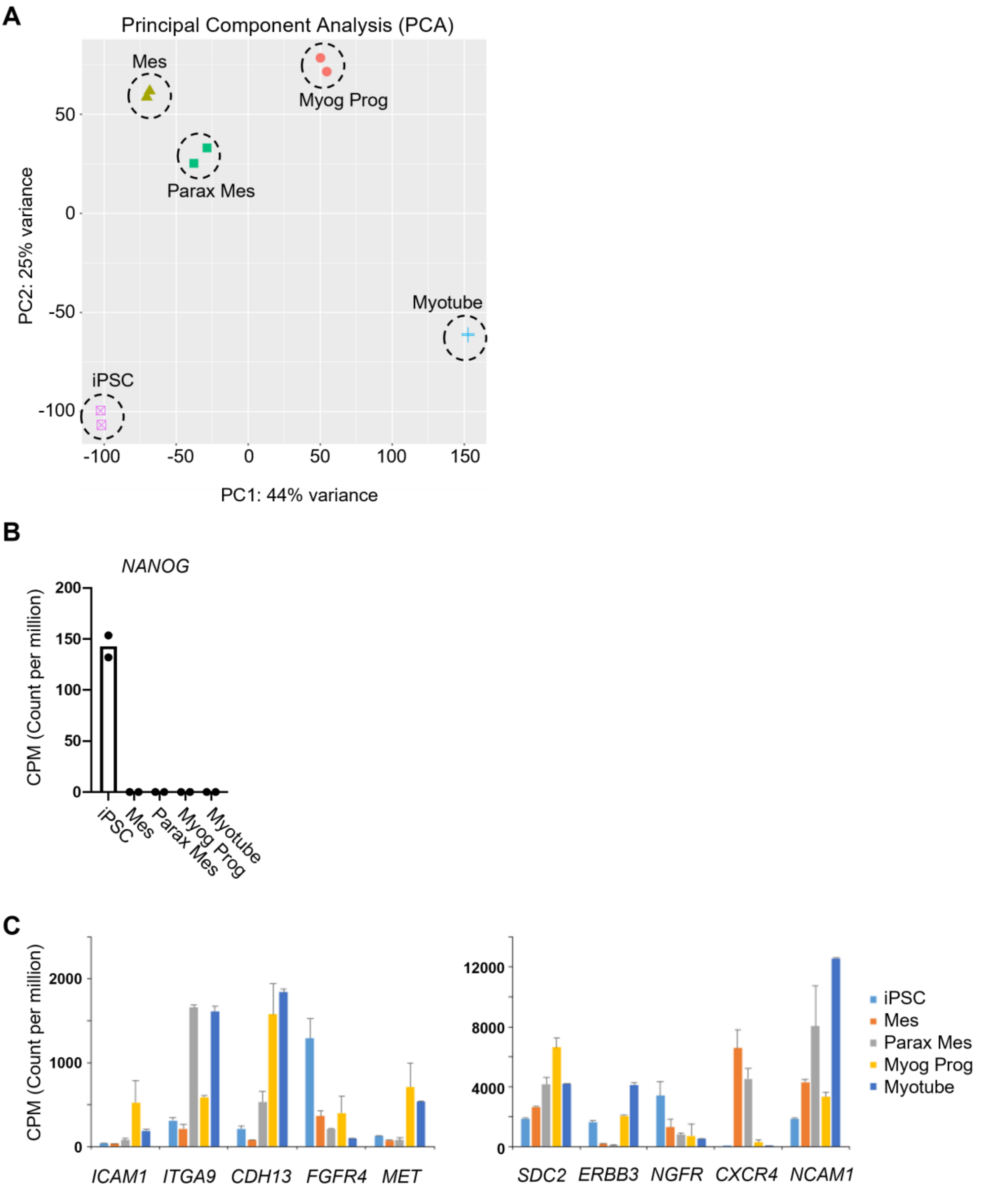

Supplementary Figure S3. Transcriptomic profiling of differentiating NHP iPAX7 iPS cells. A) Principal component analysis of differentially expressed genes identified by RNA-seq. The variability of the data set along PC1 is 44% and PC2 is 25%. B) Expression of *NANOG* across the clusters identified in the heatmap from Figure 2A. C) Expression of previously reported markers used for prospective isolation of iPSC-derived myogenic cells across the clusters identified in the heamap from

Figure 2A. *CXCR4*, *MET*, *ICAM1*, *ITGA9*, *SDC2*, *ERBB3*, *NGFR*, *CDH13*, *FGFR4*, *NCAM1*. Expression data for *CD10* and *CD24* are not available. CPM: Counts per Million Reads.

Supplementary Figure S4

A

	NHP WT
CDC crossmatch	
Lysis (%)	20
Flow crossmatch	
IgG MFI (fold change)	1.14
IgM MFI (fold change)	0.97

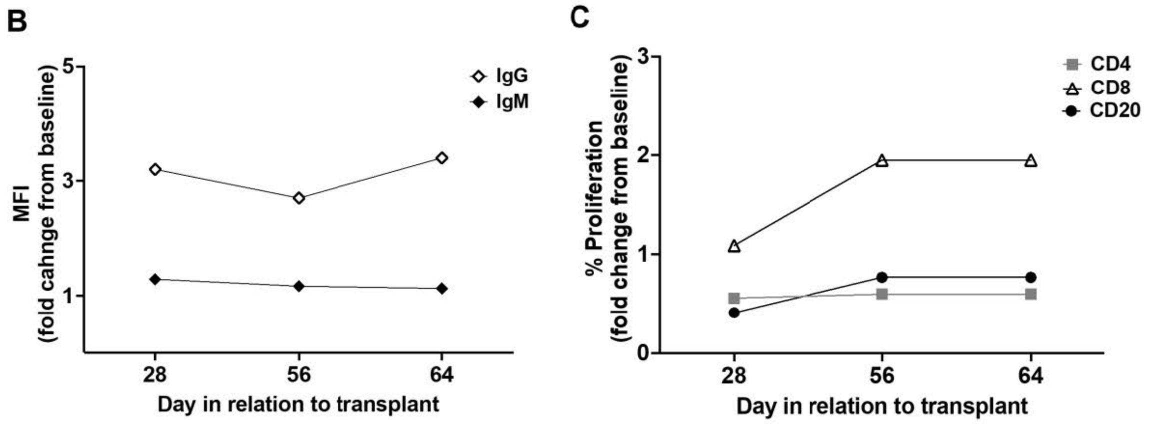

Supplementary Figure S4. Pre- and post-transplant immune monitoring. A) Crossmatching of the candidate NHP recipient was performed using wild type (WT) cell line. Complement-dependent-cytotoxicity (CDC) was used to assess the presence of preformed donor-specific antibodies, as indicated by a cytolytic reaction with a <25% lysis cutoff for enrollment. Similarly, binding of donor specific antibody to the donor cells was measured as mean fluorescence intensity (MFI) using multiparametric flow cytometry with a cutoff of 3 fold for IgG and 6 fold for IgM MFI ratios for enrollment. B) Serum levels of IgG and IgM in recipients throughout the experiment. IgG and IgM levels are represented as relative MFI (fold increase from pretransplant baseline) to illustrate the development of elicited IgG antibody. C) Analysis of cellular immune response showed a low

number of proliferating CD4 + T cells and CD20 + B cells, with a marked increase in CD8 + T cells in circulation that were reactive to myogenic progenitor cells.

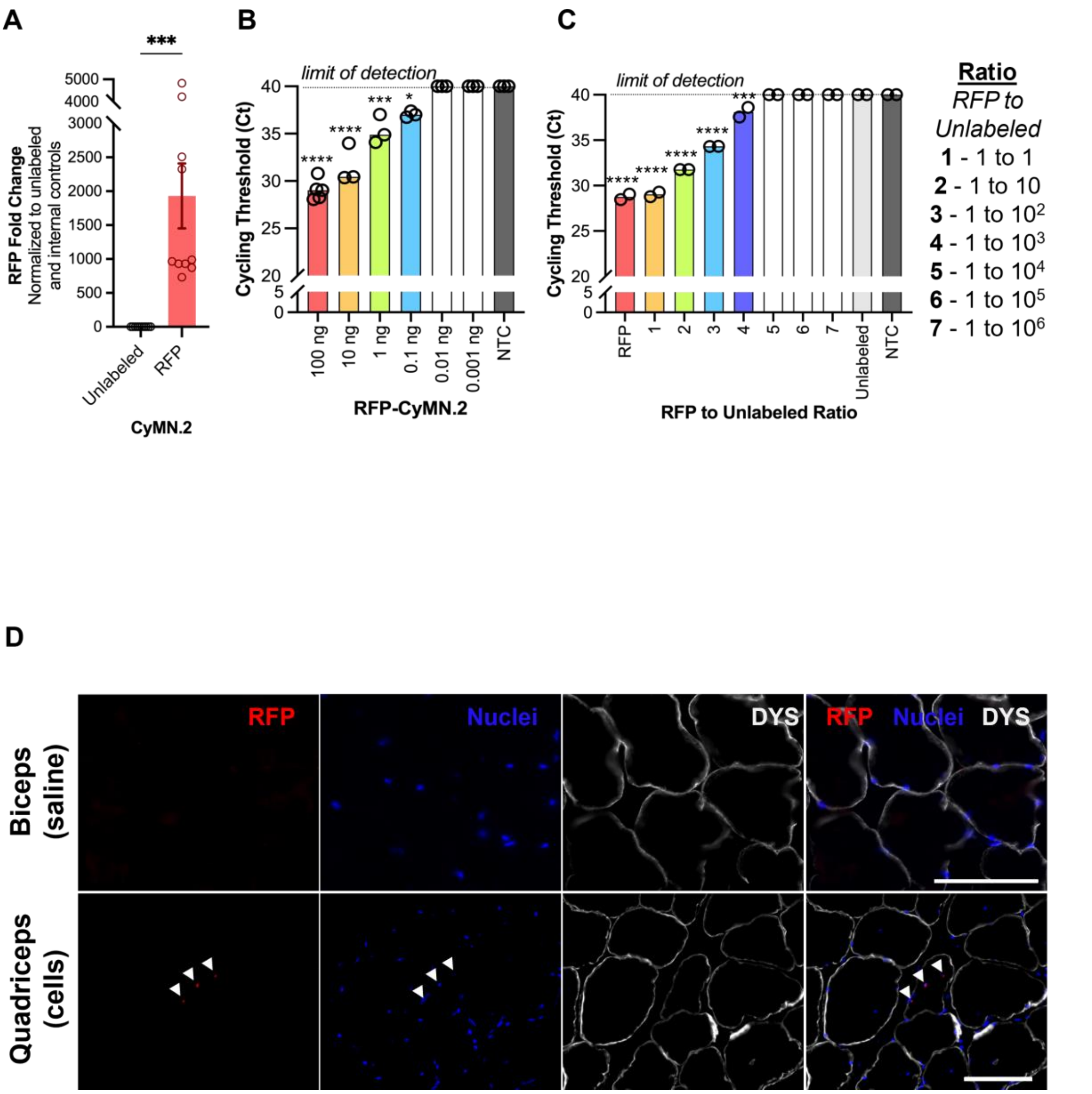

Supplementary Figure S5. Sensitivity test of RFP signal for RFP-labeled CyMN.2 PAX7+ myogenic progenitors in NHP recipient. A) Dot-plot depicting fold change of RFP signal detected in RFP-labeled or unlabeled CyMN.2 cells, normalized to β-actin internal control. Each dot represents one technical replicate. n = 3 independent replicates, with 3-4 technical replicates each. ***P=0.0008 when an unpaired t-test was performed. B) Dot-plot depicting the cycling threshold (Ct) values for RFP when CyMN.2-RFP DNA was serially diluted. The limit of detection is 0.1 ng of RFP^{+} DNA per

reaction. n = 3-5 technical replicates. One-way ANOVA, with Dunnett's multiple comparisons tests where samples were compared against NTC control. ****P<0.0001 for 100 ng and 10 ng. ***P=0.0003 for 1 ng and *P=0.0169 for 0.1 ng. P>0.99 for all others. C) Dot-plot depicting the cycling threshold (Ct) values for RFP when RFP-labeled CyMN.2 iPAX7 myogenic progenitors were mixed with unlabeled (RFP-) CyMN.2 cells at various ratios. The sensitivity for this assay is 1 RFP^+ cell in 1,000 RFP^- cells. n = 2 independent replicates, with 3-5 technical replicates each. One-way ANOVA, with Dunnett's multiple comparisons tests where samples were compared against NTC control. ****P<0.0001 for RFP+, 1, 2, 3, and 4. P>0.99 for all others. Error bars depict mean with standard error of the mean (s.e.m) for all.
